# Ten simple rules for documenting scientific software

**DOI:** 10.1371/journal.pcbi.1006561

**Published:** 2018-12-20

**Authors:** Benjamin D. Lee

**Affiliations:** School of Engineering and Applied Sciences, Harvard University, Cambridge, Massachusetts, United States of America; Dassault Systemes BIOVIA, UNITED STATES

## Introduction

Science, and especially biology, is increasingly relying on software tools to enable research. However, if you are a biologist, you likely received no training in software development best practices. Because of this lack of training, scientific software often has minimal or even nonexistent documentation, making the lives of researchers significantly harder than they need to be, with precious research time being spent figuring out how to use poorly documented software rather than performing the actual science. As the field matures, software documentation will become even more important as software stops being maintained and original authors are unable to be reached for support. Prior work has focused on various aspects of open software development [1–7], but documenting software has been underemphasized. I present these 10 simple rules in the hope that, by applying software engineering best practices to research tool documentation, you can create software that is maximally usable and impactful.

## Rule 1: Write comments as you code

Comments are the single most important aspect of software documentation. At the end of the day, people (yourself included) need to be able to read and understand your source code. Good variable and function names can help immensely with readability, although they are no complete replacement for comments. Although it may be perfectly obvious to you what your code does without comments, other readers will likely not be so fortunate. Indeed, you yourself may not even be able to understand your own code after you've moved on to another project. Think of comments as your lab notebook: they help you remember your train of thought long after the fact.

The best way to write comments is to do it as you code. That way you never have the problem of forgetting what your thought process was, and you never forget to go back and write the comments that you promised yourself you'd do (we're all guilty of this). Modern integrated development environments (IDEs) will often automatically generate documentation strings as you write code, which removes the burden of having to remember to write comments. One common argument against thorough code commenting is that it slows you down. In fact, good commenting can help you write code faster because you have a better understanding of your software. This understanding is especially useful when you run into bugs because you can compare what your code is doing to what your comments say it should be doing. Don’t forget that, at the end of the day, your code has the final word on what your software will do, not your comments.

Proper code commenting is as much an art as it is a science. If you write too few comments, people won't be able to figure out what your code is doing. Write too many and readers will get lost in the sea of comments [4]. As a guiding principle, aim to write code that readers can understand purely by reading your comments [7]. If you remember one thing from this section, when in doubt, err on the side of more comments.

To get a feel for the right amount of commenting for code, let’s examine some examples.

Bad (no comments):

for sequence in parsed_sequences:

        analyze(sequence)

Bad (too much commenting):

# iterate over the genes in the genome

for sequence in parsed_sequences:

        # call the analyze function, passing it each gene as its argument

        analyze(sequence)

Good (just enough):

# analyze the genome

for sequence in parsed_sequences:

        analyze(sequence)

The key takeaway here is to keep your comments in the Goldilocks zone—not too many and not too few.

## Rule 2: Include examples (and lots of them)

When it comes to software documentation, showing takes precedence over telling. There are several important reasons to include examples in your documentation beyond simple instruction. Examples provide a starting point for experimentation. By starting from a piece of code that works, your users can attempt to change it for their own uses with minimal difficulty.

Unlike with comments, there isn’t such a thing as too many examples if they all show off different aspects of your software. If you find that your main documentation is getting too laden with examples, feel free to move them to a special section or directory so long as you keep your examples organized and easily discoverable. Keras, a machine learning framework, has 35 full example scripts as of the time of this writing (github.com/keras-team/keras/tree/master/examples) with a README (see Rule 4 for more) explaining what each example demonstrates. Although you are by no means under any obligation to provide that many examples, do take the time to at least write examples showing off the main functionality of your software [2]. You can even make your examples do double duty as unit tests (or vice versa), thereby verifying functionality while providing instruction.

## Rule 3: Include a quickstart guide

Going from idea to experimentation to results as quickly as possible enables the progress of science. If people must spend a long time figuring out how to use your software, they're likely to give up. Conversely, if people can immediately start playing with your tool, they're vastly more likely to use it as a part of their research. It is therefore crucial to include a quickstart guide aimed at helping people begin using your software as quickly as possible.

This can take the form of an example (see Rule 2), a tutorial, a video, or anything else you can imagine. For example, let’s look at the TPOT machine learning tool’s quickstart guide [8]: it has an animated graphic image file (GIF) showing the software’s functionality, diagrams explaining how it works, and a minimal code stub, perfect for copy-pasting into your own project. To tell whether your quickstart guide is working as intended, show it to someone who hasn't used your software and see if they can figure out how to start using it. Consider your quickstart guide to be a dating profile for your project: it should show off its strengths, give people a feel for it, and entice people into choosing it.

## Rule 4: Include a README file with basic information

Your README file acts like a homepage for your project. On code-sharing sites like GitHub, Bitbucket, and GitLab, your README file is shown on your project's main page. README files should be easily readable from the raw source, so human-readable markup languages such as Markdown or reStructuredText (or plain text) are preferable to less readable formats like hypertext markup language (HTML). In fact, code-sharing sites will usually render your markup language on your repository's page, giving you the best of both worlds. Take advantage of this—free hosting is hard to come by and the fact that your hosted README page is on your repository makes the arrangement even sweeter.

A good rule of thumb is to assume that the information contained within the README will be the only documentation your users read. For this reason, your README should include how to install and configure your software, where to find its full documentation, under what license it’s released, how to test it to ensure functionality, and acknowledgments. Furthermore, you should include your quickstart guide (as introduced in Rule 3) in your README.

Often, the top of your README files will include badges that, when rendered, show the status of the software. One common source of badges is shields.io, which can dynamically generate badges for your project. Common badges include ones that show whether automated tests are passing (such those from travis-ci.org), what percentage of the code that the tests cover, whether the documentation is up to date, and more. Although not necessary, these badges instill confidence in the quality of your project and convey important information at a glance and are therefore highly recommended.

## Rule 5: Include a help command for command line interfaces

Many scientific software tools have command line interfaces (CLIs). Not having a graphical interface saves development time and makes the software more flexible. However, one challenge that CLI software has is that it can be hard to figure out how to use. The best way to document CLIs is to have a “help” command that will print out how to use the software. That way, users don't need to try to find your documentation to get basic tasks done. It should include usage (how to use the command), subcommands (if applicable), options and/or arguments, environment variables (if applicable), and maybe even some examples (Rule 2 strikes again!).

A help command can be tedious to make and difficult to maintain, but luckily there are numerous software packages that can do it for you. In Python, software such as click (click.pocoo.org) can not only make your help command but can also even help you make your interface, saving you time and effort.

An example of a good CLI is the one included in Magic-BLAST. It has a short help command, “-h,” which provides basic information on what the tool is and how to use it. It also includes instructions on how to access the full help documentation, which include a list of every option as well a description of the option’s arguments and what it does. An arrangement like this is particularly good because it requires minimal effort to find just the most useful information via the short help page, thereby reducing information overload and reducing the cognitive load of using the software by providing a reminder of how to access the full CLI reference.

## Rule 6: Version control your documentation

A previous Ten Simple Rules article has described the virtues of using Git for your code [1]. Because your documentation is such an integral part of your code, it must be version controlled as well. To start, you should keep your documentation inside your Git repository along with the rest of your files. This makes it possible to view your documentation at any point in the project's history. Services such as Read the Docs (readthedocs.org) and Zenodo (zenodo.org) make doing this even easier because they will archive a complete rendered version of your documentation every time you make a new release of your software.

To illustrate why this is such an important rule, consider what would happen if you change a default setting in a new release of your software. When users of previous versions go to look at your documentation, they will see the documentation that is incompatible with the version that they have installed. Worse still, because you changed a default, the software could fail inexplicably. This can be incredibly aggravating to users (and even dangerous if the software is for mission-critical applications), so it is extra important to use version control for your documentation. A changelog in your documentation can make this task much easier. If you are using informative commit messages, creating a changelog is a straightforward task at worst and a trivial task at best.

As an example of a bioinformatics library that is doing a particularly good job at version controlling their documentation, look at khmer, which has a thorough changelog containing new features, fixed bugs (separated by whether they are relevant to users or developers), known issues, and a list of the contributors to the release [9]. In addition, previous versions of the documentation website are easily accessible and labeled clearly. By providing this information, the authors have ensured that users of any version of the software can get the right version of the documentation, see what’s going on in the project, and make sure they’re aware of any issues with their version.

If you take one thing away from this rule, make it very clear which version of your software your documentation is for and preserve previous versions of your documentation—your users will thank you.

## Rule 7: Fully document your application programming interface

Your application programming interface (API) is how people who are using your software interact with your code. It is imperative that it be fully documented in the source code. In all honesty, probably nobody will read your entire API documentation, and that's perfectly fine. The goal of API documentation is to prevent users from having to dig into your (well commented, right?) source code to use your API. At the very least, each function should have its inputs and input types noted, its output and output type noted, and any errors it can raise documented. Objects should have their methods and attributes described. It’s best to use a consistent style for your API documentation. The Google style guide (google.github.io/styleguide) has API documentation suggestions for numerous languages such as Python, Java, R, C++, and Shell. You spent a lot of time developing your API; don’t let that time go to waste by not telling your users how to use it.

## Rule 8: Use automated documentation tools

The best type of documentation is documentation that writes itself. Although no software package can do all your documentation for you (yet), there are tools that can do much of the heavy lifting, such as making a website, keeping it in sync with your code, and rendering it to a portable document file (PDF). Software such as Sphinx (sphinx-doc.org), perldoc, Javadoc, and Roxygen (https://github.com/klutometis/roxygen) for R can generate documentation and even read your comments and use those to generate detailed API documentation. Although Sphinx was developed to host Python’s documentation, it is language agnostic, meaning that it can work for whatever language your project is in. Similarly, Doxygen (doxygen.nl) and MkDocs (mkdocs.org) are language-agnostic documentation tools. Read the Docs, introduced in Rule 6, is a language-agnostic documentation hosting platform that can rebuild your documentation every time that you push to your repository, ensuring that your documentation is always up to date.

There are many other ways automation can make your documentation smarter: in Python, software like doctest (sphinx-doc.org/en/stable/ext/doctest.html) can automatically pull examples from your documentation and ensure that your code does what you say it should be doing in the documentation. To help you follow Rule 7, there are tools such as Napoleon (github.com/sphinx-contrib/napoleon) that can generate your API documentation for you. It’s even possible to automatically generate interactive representational state transfer (REST) API documentation using free tools such as Swagger (swagger.io). At this point, there is almost no reason not to be using automated documentation tools.

## Rule 9: Write error messages that provide solutions or point to your documentation

Error messages are part of life when developing software. As a developer, you should be doing your best to make your error messages as informative as possible. Good error messages should have three parts: they should state what the error is, what the state of the software was when it generated the error, and either how to fix it or where to find information relevant to fixing it. In the spirit of Rule 2, let's look at an example.

Bad:

Error: Translation failed.

Good:

Error: Translation failed because of an invalid codon ("IQT") in position 1 in sequence 41. Ensure that this is a valid DNA sequence and not a protein sequence.

By showing what exactly went wrong and proposing a fix for it, your users will spend less time debugging and more time doing science. Since you know your software better than anyone else, providing guidance in error messages can be invaluable. If for no other reason, do it to save yourself the hassle of being tech support for your users (most of whom have barely read your documentation, if at all) when they run into easily fixable usage mistakes.

Furthermore, it is important to say what the state of the software was when the error was generated, especially if it takes a long time to run or you don’t save logs by default. If your software fails, seemingly at random, after 12 hours of execution, your users will be thankful to know what was going on when the error was thrown rather than having to wait another 12 hours to reproduce the error with logging enabled.

## Rule 10: Tell people how to cite your software

Of all the rules in this guide, odds are that this is the one you need the least. However, it must be said that, if you publish scientific software, you need to include the information required to properly provide attribution to your work. I recommend providing the digital object identifier (DOI), a BibTeX entry, and a written reference for your publication in your README, as well as using a “CITATION” file in citation file format (CFF) format, which is a human- and machine-readable file format designed for specifying citation information for scientific software [10].

Including citation information in your documentation is especially important for software that has not been published in a traditional academic journal, which would assign it a DOI. Just because your software is unpublished doesn't mean that you can't get a DOI for it—you deserve credit for your work. If you're using Zenodo to archive your releases (see Rule 6), it will mint a new DOI for each release as well as a DOI for the entire project. Another great, free way to get a DOI for your project is to submit it to the Journal of Open Source Software (joss.theoj.org), a peer-reviewed open-access academic journal designed for software developers. Both even provide a badge for your README (see Rule 4) so that the entire world can tell how to cite your software at a glance.

## Conclusion

I hope that this guide will help you improve the quality of your software documentation. Documenting software is not always as exciting as doing original software development, but it is nonetheless as important. Software documentation is in many ways like writing a paper in that it is a required step in the dissemination of your ideas. It is a critical step for ensuring reproducibility, not to mention the fact that many bioinformatics journals now require that software submitted be well documented. Automated documentation tools such as Sphinx can diminish some of the effort required for good documentation, perhaps even making the work enjoyable. Finally, because documentation can make or break a project’s adoption in the real world, by following these 10 simple rules you can give your project its best chance of wide adoption and possibly even end up as an example of good documentation in a Ten Simple Rules article!
